# A Knowledge-Based Machine Learning Approach to Gene Prioritisation in Amyotrophic Lateral Sclerosis

**DOI:** 10.3390/genes11060668

**Published:** 2020-06-19

**Authors:** Daniel M. Bean, Ammar Al-Chalabi, Richard J. B. Dobson, Alfredo Iacoangeli

**Affiliations:** 1 Department of Biostatistics & Health Informatics, King′s College London, 16 De Crespigny Park, London SE5 8AF, UK; richard.j.dobson@kcl.ac.uk; 2 Health Data Research UK London, University College London, 16 De Crespigny Park, London SE5 8AF, UK; 3 King′s College Hospital, Bessemer Road, Denmark Hill, Brixton, London SE5 9RS, UK; ammar.al-chalabi@kcl.ac.uk; 4 Maurice Wohl Clinical Neuroscience Institute, Department of Basic and Clinical Neuroscience, King′s College London, London, 5 Cutcombe Rd, Brixton, London SE5 9RT, UK; 5 Institute of Health Informatics, University College London, 222 Euston Rd, London NW1 2DA, UK

**Keywords:** gene prioritisation, machine learning, gene discovery, amyotrophic lateral sclerosis, motor neurone disease, knowledge graph

## Abstract

Amyotrophic lateral sclerosis is a neurodegenerative disease of the upper and lower motor neurons resulting in death from neuromuscular respiratory failure, typically within two to five years of first symptoms. Several rare disruptive gene variants have been associated with ALS and are responsible for about 15% of all cases. Although our knowledge of the genetic landscape of this disease is improving, it remains limited. Machine learning models trained on the available protein–protein interaction and phenotype-genotype association data can use our current knowledge of the disease genetics for the prediction of novel candidate genes. Here, we describe a knowledge-based machine learning method for this purpose. We trained our model on protein–protein interaction data from IntAct, gene function annotation from Gene Ontology, and known disease-gene associations from DisGeNet. Using several sets of known ALS genes from public databases and a manual review as input, we generated a list of new candidate genes for each input set. We investigated the relevance of the predicted genes in ALS by using the available summary statistics from the largest ALS genome-wide association study and by performing functional and phenotype enrichment analysis. The predicted sets were enriched for genes associated with other neurodegenerative diseases known to overlap with ALS genetically and phenotypically, as well as for biological processes associated with the disease. Moreover, using ALS genes from ClinVar and our manual review as input, the predicted sets were enriched for ALS-associated genes (ClinVar *p* = 0.038 and manual review *p* = 0.060) when used for gene prioritisation in a genome-wide association study.

## 1. Introduction

Amyotrophic lateral sclerosis (ALS) is a neurodegenerative disease of the upper and lower motor neurons resulting in death from neuromuscular respiratory failure, typically within 2–5 years of the first symptoms [1]. Genetic factors are an important cause of ALS, and over 130 genes [2] have been linked to the disease, although only a subset have strong and replicated supporting evidence [3]. Rare disruptive variants are responsible for about two-thirds of familial cases [4] and 10–15% of the remaining 90–95% of patients who do not report a family history of ALS [4,5,6]. Therefore, for most people with ALS, the cause of the disease remains unexplained. Gaining a better insight into the disease genetics could lead to the development of new treatments and, ultimately, a cure.

With the advance of high-throughput technologies, we now have large amounts of protein–protein interaction and phenotype-genotype data available [7,8]. Although such big data offer great potential for the study of human diseases, their use presents significant challenges, including the interpretation and prioritisation of the results [3,9]. Studies have shown that genes involved in specific or related diseases tend to be located in neighbouring regions of the protein–protein interaction network [10], and their interactions often follow similar network patterns [11]. Machine learning methods, such as graph-based methods, are well-positioned to exploit this valuable source of information for candidate-gene prioritisation. 

Accordingly, many methods to date have been developed and applied to predict new candidate disease-associated genes based on prior biological knowledge and phenotype information. Many use graph-based methods, the most widely used being diffusion or random walk algorithms [12,13,14,15,16,17]. Other graph-based approaches include N2VKO [18], using node embeddings based on node2vec [19], CIPHER [20], based on gene network closeness and phenotype similarity, a gene gravity-like algorithm [21], and deep learning [22]. Other approaches not based on graphs include machine learning algorithms trained on functional similarity [23]. In this work, we explored the use of our in-house machine learning method [24] that, given a prior definition of the genes linked with the disease, exploits the phenotypic and biological information from publicly available databases, to predict new candidate ALS genes. We explored how the initial definition of the known ALS-linked gene set impacts results. We also investigated the relevance of the predicted putative ALS genes by studying their role in other diseases known to be related to ALS, the biological processes they are involved in, and by using the results from the most recent ALS genome-wide association study (GWAS) for which summary statistics were publicly available.

## 2. Materials and Methods

### 2.1. Data Sources

The disease-gene prediction was based on protein–protein interaction data from IntAct [25], gene function annotation from Gene Ontology [26,27] release 2019-07-01, and known disease-gene associations from DisGeNet v6.0 [28]. The edge was placed between the genes that encode each protein, to model the protein–protein interactions.

### 2.2. Definition of ALS-Linked Genes

In total, five sets of ALS-linked genes were used in this study: (1) DisGeNet—101 genes [28], (2) ALSoD—126 genes [2,29], (3) ClinVar—44 genes [30], (4) a manually-curated list—40 genes [3] and (5) the union of all other sets—199 genes. DisGeNet contains several specific subtypes of ALS as UMLS identifiers. For this study, we chose to merge these subtypes to prevent the presence of very similar diseases making the learning unrealistically easy. Gene lists are available as Supplementary File 1. Note that while multiple definitions of ALS-linked genes were used, all other disease-gene associations were from DisGeNet. 

### 2.3. Machine Learning

We used our recently published method for knowledge graph completion (available at https://github.com/KHP-Informatics/ADR-graph) (version 0.1) [24]. In brief, the method takes as input a graph of known data related to the prediction task, in this case, gene-disease links, gene functions, and others, and returns a list of predicted edges missing from that graph. These relationships are predicted to exist based on similar patterns found in the graph. For example, to predict new genes linked to ALS, the algorithm compares all genes known to be linked to ALS to all other genes and builds a predictive profile based on a weighted combination of existing relationships in the graph. Every gene is then scored for its similarity to this profile. Predictions are made by applying a threshold to this similarity score, with all genes above the threshold predicted as candidate ALS-linked genes. The optimum weighting and score threshold are learned from the known set of linked genes [24]. 

### 2.4. Cross-Validation

The goal of the machine learning algorithm is to predict new genes linked to ALS based on facts about the genes known to be associated. In the cross-validation, this task is simulated by deleting a proportion of the known genes and training the algorithm on the remaining data before testing whether it can predict the link to the missing genes correctly. Unlike a typical machine learning cross-validation, where the training and test sets are entirely separate, here, the algorithm was trained on the full set of genes, but the link to ALS of the test set was deleted from the graph. This means that during training, the “missing” linked genes were labelled as not linked to ALS. Therefore, the job of the algorithm was to replace this missing link in the graph, making the task harder as genes that were linked to ALS were included during training as true negatives. This is an exact simulation of the use-case. 

### 2.5. Gene Set Function and Phenotype Enrichment Analyses

Gene ontology term overrepresentation was analysed using Pantherdb [31] version 15.0. All tests were performed using the “Panther GO slim for biological process” and the Fisher′s exact test for overrepresentation with the Bonferroni correction for multiple testing. Enrichment vs. the background list with *p*-value < 0.05 after the Bonferroni correction was considered significant. Similar biological processes were grouped with Revigo [32]. For the predicted ALS-linked gene sets, the genes in the knowledge graph that were not linked to ALS according to the relevant definition were used as the background gene list (i.e., the set of all genes that could have been predicted).

We used the Enrichr webserver [33] to test our predicted gene lists for the enrichment of genes linked to human diseases in the OMIM database [34,35]. An adjusted *p*-value < 0.05 was considered significant.

### 2.6. GWAS Validation

To validate the association between the candidate genes proposed by our method and ALS, we used the largest ALS GWAS for which summary statistics were publicly available at the time [36]. It included 80,610 individuals (20,806 cases and 59,804 controls) of European ancestry. For each set of genes, only SNPs mapped onto those genes were used for validation. The single-test *p*-value threshold to indicate statistical significance was 0.05. When multiple tests were performed, we used the Bonferroni correction [37] based on the number of genes being tested. All annotations, genomic positions, and variants refer to the reference human genome hg19/GRCh37. The 1000 Genomes Project Phase 3 reference panel was used to compute the *r^2^* and MAF [38]. The gene-based and gene-set association analyses were performed with Magma v1.07b [39]. In the gene-based and gene-set analyses, the associations of all SNPs within each gene or gene-set were tested simultaneously. 

## 3. Results

### 3.1. Definition of Known ALS-Linked Genes

**Figure 1:**
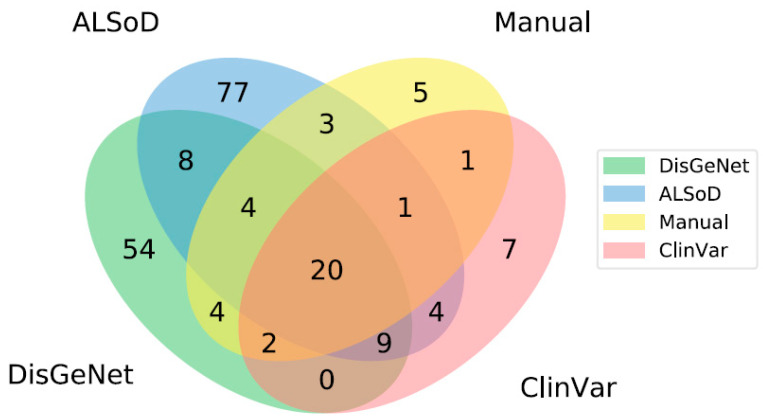
Overlap between lists of ALS-linked genes from different sources. Note that areas are not to scale. There are no genes that are listed in both ClinVar and DisGeNet but not found in either ALSoD or the manual review. In total, there are 199 unique genes linked to ALS across all lists.

Given that there is no consensus on what genes increase the risk of ALS, we chose to separately model lists of genes from a range of sources (Supplementary File 1). These included a specialized public database of ALS gene mutations in humans, the ALS Online Database (ALSoD), a general database of clinical relevant mutations, ClinVar, a general database of all disease-associated (in the broadest sense) genes, DisGeNet, our manual review of ALS-linked genes curated by scientists working in the field, and the union of these four lists. The overlap between these lists is shown in Figure 1. 

### 3.2. Model Training and Cross-Validation

We trained a model for each of the sets of ALS-linked genes separately and used 5-fold cross-validation to estimate the performance of each predictive model (Table 1). The purpose of the cross-validation was to estimate how well a model trained on all known ALS-linked genes would perform in predicting novel associations, by testing whether it would have predicted some of the known genes based on the rest. 

For all lists, the training precision was low, which was expected for this task as the validation genes remained in the graph and were considered true negatives during training. Ideally, the model should have labelled these as positives (which would be considered “false positives” by standard evaluation metrics). These “false positives” were taken as a prediction that we subsequently validated. The performance of the DisGeNet list was notable. Although the precision was relatively high, the standard deviation was also much greater. This indicated that performance was very sensitive to randomisation of the folds, possibly due to a less uniform set of genes in terms of the available data. The model also achieved much lower recall for DisGeNet (only 23% in training and 9% in validation), and only 2/5 training folds were significantly enriched for validation. The lower recall of the DisGeNet list could be due to DisGeNet being a broad database, with no manual curation that also includes genes linked to ALS only in model organisms. Models based on all other lists significantly outperformed random guessing in all folds, indicating the model has learned discriminative features of the provided ALS-linked genes. 

### 3.3. Prediction of New ALS-Linked Genes

**Figure 2:**
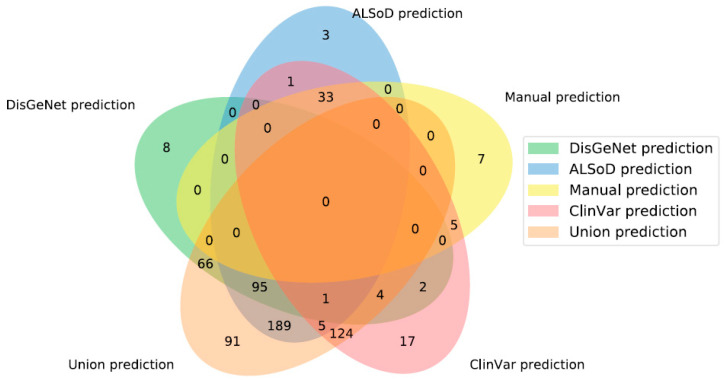
Overlap of predicted ALS-linked genes from the five gene lists. “Union prediction” is the new predictions made, based on the union of all other lists of known linked genes, not the union of all predictions. Note that areas are not to scale.

For each set of ALS-linked genes, we trained a model on the full list and generated new predictions based on the learned profile. Taking the full set of new ALS-linked genes predicted from each list gave 45 (Manual list), 176 (DisGeNet), 192 (ClinVar), 327 (ALSoD), and 575 (union) predicted linked genes (Figure 2). The genes predicted by the model trained on the manually curated list are shown in Table 2. The full set of predicted genes from all models are available in Supplementary File 2.

As shown in Figure 2, there were no genes predicted by all definitions of known ALS-linked genes. The main reason for this is that the input lists are not identical and frequently predict members of other lists. For example, a prediction made from ALSoD cannot be predicted from DisGeNet if it is already linked to ALS in the DisGeNet list. Moreover, 91 genes were uniquely predicted by the union set, meaning that this list did not simply produce the union of the outputs of all other lists. 

**Figure 3:**
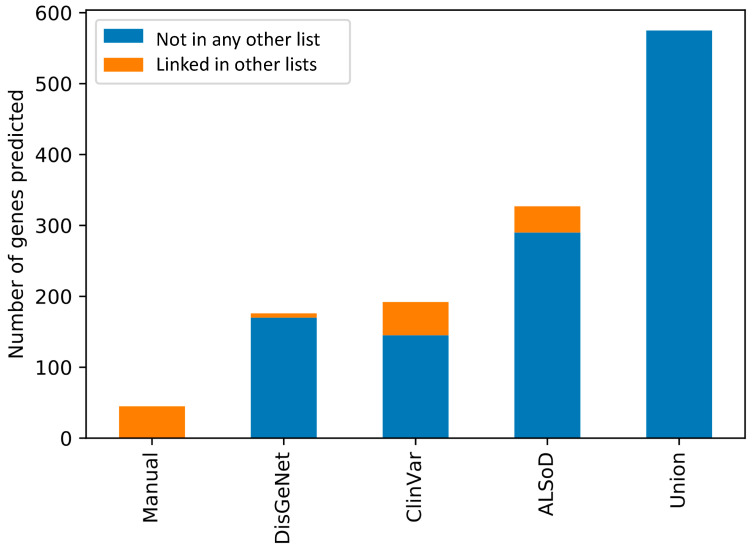
Overlap of the predicted ALS-linked genes from each input list with the other lists of known genes. All the predictions from the Manual list are already contained in at least one other list. By definition, none of the predictions from the union list are in any other list.

In contrast, 15 genes were predicted from at least one other list but not the union list and which were not part of any list themselves. Although there are 73 genes outside of the union predictions in Figure 2, many were linked to ALS in other lists. In other words, combining all lists, and then training the model is not the same as training a model on each list and then combining the results. This is likely due to the impact of combining lists of genes on the enrichment test in the feature selection step of model training. Figure 3 shows the number of predictions from each list that were already considered to be linked to ALS in at least one other list. 

The 45 genes predicted from the Manual list of ALS-linked genes were all contained in at least one other list. All 45 were in DisGeNet, 12 were in ALSoD, and seven were in ClinVar. 

### 3.4. Functional and Phenotypic Enrichment Analyses of Predicted ALS-Linked Genes

Given that our trained models were significantly enriched for ALS-linked genes in a simulation of this task (the cross-validation), the new predictions from the full models, using the whole lists as training datasets, might contain new ALS-linked genes. The predicted sets of genes overlapped to varying degrees and could be functionally similar. We used GO term overrepresentation applied to each set of predicted genes to investigate which biological processes they were enriched for. A complete list of significantly enriched processes and corresponding GO terms is available in Supplementary File 3. All predicted sets were enriched for genes involved in biological processes associated with ALS. For example, considering the 20 processes with the highest enrichment fold for practicality (Table 3), several processes related to angiogenesis [40], lipid metabolism [41], mitochondria activity [42], protein kinase activity [43], superoxide metabolism [44,45], vesicle-trafficking [46], neurotransmitter regulation [47], and behaviour [48] were widely shared across the sets.

We also investigated whether the sets of predicted ALS-linked genes were enriched for genes associated with other related neurodegenerative diseases. In this test, we used Enrichr with the disease-gene sets from the OMIM database (Table 4). Several neuromuscular diseases, e.g., Dystonia and Cardiomyopathy, and neurodegenerative disorders, e.g., Charcot-Marie-Tooth disease (CMTD), Parkinson′s disease (PD), Frontotemporal dementia (FTD), Schizophrenia (SCZ) and Alzheimer's Disease (AD), were among the top 5 significant hits (Table 4). For some of these diseases, e.g., FTD, SCZ, PD, and CMTD, the genetic and phenotypic overlap with ALS has been shown previously [49,50,51,52,53,54,55]. Interestingly, type 2 diabetes was among the top 5 DisGeNet hits and has been proposed as an ALS risk factor and phenotypic modifier [56,57]. The predictions from the Manual list were enriched for genes already linked to ALS in the OMIM database, again highlighting the importance of using different gene lists.

### 3.5. Validation of Predicted ALS-Linked Genes in GWAS Data

In GWAS, multiple testing correction approaches, such as the Bonferroni method, are used to protect from Type I errors. However, at a genome-wide scale, the price to pay for a minimal Type I error is the limitation of the statistical power provided by the sample. One way to overcome this issue is to restrict the number of tests to a limited number of candidate genes, such that a less stringent *p*-value threshold can be used. To test the relevance of the predicted genes in ALS further, and explore the feasibility of using our method to prioritise GWAS hits, we used our predicted gene sets to identify new ALS genes based on a previously published ALS GWAS. This study involved over 80,000 individuals of European ancestry, about a quarter of whom were people with ALS and the remainder unaffected controls. 

Using the publicly available summary statistics, we performed a Magma gene-based analysis that combined all SNPs within each gene to test their association with ALS simultaneously. The ~10,000,000 SNPs from the GWAS were mapped onto 18,067 protein-coding genes. For each list, a *p*-value smaller than 0.05/N was used to indicate a significant association. N was the number of genes in the set that were effectively tested in the Magma gene analysis. At least one new prediction was validated for each model by this method (Table 5). However, when we tested the likelihood of our findings occurring by chance, only the ClinVar model (*p* = 0.038) passed the significance threshold, while the Manual model was close (*p* = 0.060). 

*ATXN3* was predicted and validated for three models and *WNT7A* for two models. For the *ZFP91-CNTF* locus, the whole co-transcript, including both *ZFP91* and *CNTF* was used instead of the two individual genes, as the SNPs within these two adjacent genes were in strong linkage disequilibrium (Supplementary Figure S1). Note that even if more than one model predicted the same gene, it would not have necessarily been validated by all of them as the *p*-value threshold depended on the total number of genes predicted by each model. The Magma gene-set analysis allowed us to test all the genes within each list simultaneously. Similar to the single-gene analysis, the Manual and ClinVar models were the ones with the lowest *p*-values (*p* = 0.057 and *p* = 0.065, respectively). 

## 4. Discussion

In this study, we trained machine learning models to predict new ALS genes based on multiple available definitions of the currently known ALS genes. Overall, all models performed well in simulations and were able to predict genes involved in ALS-related diseases and biological processes. Only one of the five models (based on ClinVar) produced significant results (*p* = 0.038) when validated in human GWAS data. 

There are several databases of disease-gene associations, as well as several literature reviews, but there is no consensus list of all currently known ALS-linked genes. Partly, this is because ALS is a complex, rare, and rapidly progressive disease whose study involves elaborate designs that are difficult to implement and replicate; partly because such lists and databases utilise different definitions of ALS-linked genes according to their aim. For example, we used a manually curated list of genes containing only genes with strong and replicated evidence of association with ALS. In contrast, large non-specialist databases might consider weaker evidence such as non-replicated differential expression in a transgenic animal model as sufficient evidence. Neither of these examples is necessarily correct or incorrect, but the choice of genes is very likely to impact the results of a predictive study such as this, where predictions are based on similarity to the genes that we use to define ALS. Our choice to build four models based on four different definitions of ALS genes and a fifth model based on the union of the four gene lists reflects such considerations. 

We trained our models using only known disease-associated genes, protein–protein interactions, and gene functional annotations. We anticipate that the inclusion of additional data could significantly improve the performance of the models. However, care must be taken to ensure that additional data types do not inadvertently leak information. By this, we mean that the indirect relationships between nodes in the graph can imply information we intended to remove. For example, we considered the inclusion of drug-disease and drug-target data in the model, but as ALS is the only disease linked to Riluzole, this would link all ALS genes indirectly to Riluzole [58], making the edges deleted in the cross-validation, trivial to predict. Furthermore, some types of information are not independent. For example, a drug treatment might only be developed based on a known ALS gene, making the cross-validation challenging to evaluate fairly. In effect, this means the time series of events must be known so that it can be used to filter out subsequently derived information and exclude it from the graph, but this information is not trivial to access at scale for many data types.

Beyond the specific genes, our results also have important implications for the use of genetic databases in machine learning in general. We have shown that when used to train the models, the different definitions of known ALS genes derived from different data sources produced very different results, such that simply combining these definitions produced further distinct predictions. The key implication of this result is that the outputs of separate studies can only be compared with considerable caution, if at all, and future work should be very transparent as to the definitions and sources of data used. 

The major challenge for the use of this knowledge-based machine learning approach to predict new ALS genes is the validation of the predicted genes. We attempted several approaches to test the performance of the models and assess the relevance of the predictions in ALS. First, the cross-validation test showed that by using a subset of the genes in the lists, the models were able to predict a significant part of the missing data in most cases. The model cross-validation performance was higher for the Manual list, matching the expectation that the genes from a manually curated and conservative source might represent a more homogeneous and reliable description of the disease genetics. Secondly, we assessed which biological processes were over-represented in our predictions. All the predicted sets of genes were enriched for biological processes known to be affected by ALS including processes related to angiogenesis [40], lipid metabolism [41], mitochondria activity [42], protein kinase activity [43], superoxide metabolism [44,45], vesicle-trafficking [46] and neurotransmitter regulation [47]. Interestingly, behaviour related processes [48] were over-represented in the DisGeNet, ALSoD, and Union predictions. Behavioural changes are frequent in ALS patients [48] and closely related to the cognitive decline observed in a significant proportion of patients [59,60]. Thirdly, we performed a phenotype enrichment analysis to test whether our predictions were significantly enriched for genes linked to other diseases. This test showed that all models predicted a significant number of genes that are involved with other neurodegenerative and neuromuscular diseases whose genetic overlap with ALS is either known or for which there is increasing recognition. These included FTD, SCZ, PD, and CMTD. Again, the Manual predictions were the most conservative as the only other enriched disease was FTD whose genetic overlap with ALS is established, and the two phenotypes often coexist in patients [61]. 

Finally, we used our predictions in a candidate gene approach to discover new genes associated with ALS using a Magma gene analysis based on the publicly available summary statistics of the most recent ALS European GWAS. We were able to validate several associations, including some known candidate genes such as *SCFD1* [62] and *UNC13A* [62]. However, when we considered the number of validated genes per model, only for ClinVar was this significantly larger than what was expected by chance (3 genes validated out of 170, *p* = 0.038). For the Manual set, this was close to significance (2 genes out of 41, *p* = 0.060). The Manual and ClinVar sets were also the only ones close to the significance threshold in the gene-set analysis that evaluated the association of all genes in each set and the disease simultaneously (*p* = 0.057 and *p* = 0.065 respectively). It is important to acknowledge that although the SNP based GWAS offered a possibility to validate our predictions, and machine learning methods such as ours are often used for variant prioritisation in GWAS, SNPs represent a limited amount of ALS heritability (~8%) [62]. Therefore, we expected a limited number of genes to be validated in this test. Given that a larger part of ALS heritability is due to rare gene mutations [62], we considered using a rare variant GWAS for validation, but the lack of publicly available summary statistics of well-powered studies of this kind limited this possibility.

We aimed to test the performance of our prediction method [24], previously validated for predicting drug side effects, in the context of gene discovery and prioritisation in ALS. However, we cannot claim that our method would perform better than alternative approaches as we did not perform any comparative assessment with respect to other techniques. The major difference between our method and most others is that ours is not based on a diffusion process such as random walks with restarts, and instead uses the local neighbourhood and an enrichment test to build the model. It will be important for future work to benchmark multiple methods across diseases to establish performance characteristics systematically across approaches, but that was not the aim of this work.

The genetics of ALS has proven challenging, and even though great progress has been made by generating and analysing large multi-omics datasets [36,62,63,64,65,66,67,68,69,70,71], the causes of ALS in most patients (~85%) remain unexplained. Using machine learning models to leverage our current knowledge of ALS and other diseases could allow us to accelerate our progress in the understanding of the genetic causes of ALS and lead towards new avenues of treatment. 

## Figures and Tables

**Table 1:** Cross-validation performance for each definition of ALS-linked genes. Precision, recall, and fold-change enrichment are given as the mean (standard deviation) for five folds. The numbers of genes are minimum-maximum. The fold-change enrichment is based on the expected precision of random guessing. Each of the five folds is considered significantly enriched if the *p*-value for predicting at least as many genes correctly is < 0.05 under the hypergeometric distribution.

	Training			Validation				
	# of Genes	Precision	Recall	# of Genes	Precision	Recall	Fold-Change Enrichment	Number of Significantly Enriched Folds
ALSoD	60–61	0.23 (0.16)	0.55 (0.09)	15–16	0.07 (0.08)	0.45 (0.17)	23.33 (23.53)	5/5
ClinVar	29–30	0.16 (0.09)	0.86 (0.04)	7–8	0.05 (0.02)	0.82 (0.17)	30.05 (15.64)	5/5
DisGeNet	54–55	0.44 (0.33)	0.23 (0.16)	13–14	0.15 (0.22)	0.09 (0.08)	55.90 (81.66)	2/5
Manual	21–22	0.27 (0.01)	0.85 (0.04)	5–6	0.09 (0.01)	0.86 (0.14)	84.54 (13.27)	5/5
Union	96	0.16 (0.04)	0.73 (0.05)	24	0.04 (0.02)	0.67 (0.07)	8.92 (4.28)	5/5

**Table 2:** Genes predicted to be linked to ALS by the model trained on the manually curated list. For each gene, we reported the Ensembl gene name, coordinates (hg19) of the longest transcript, strand, and whether the gene was present in the other lists of genes or among their model predictions. Please note that if a gene were present in one list, it could not be predicted by its corresponding model.

	Ensembl Gene Name	Ensembl Transcript Name	Chr	Transcript Start	Transcript End	Strand	ClinVar Genes	ClinVar Predictions	DisGeNet Genes	DisGeNet Predictions	ALSoD Genes	ALSoD Predictions
ALS2	ENSG00000003393	ENST00000489440	2	202581364	202591275	−	TRUE	FALSE	TRUE	FALSE	TRUE	FALSE
BCL2L1	ENSG00000171552	ENST00000307677	20	30252254	30310701	−	FALSE	TRUE	TRUE	FALSE	FALSE	TRUE
BSG	ENSG00000172270	ENST00000573216	19	572571	581376	+	FALSE	TRUE	TRUE	FALSE	FALSE	TRUE
CASP1	ENSG00000137752	ENST00000436863	11	104896234	104905977	−	FALSE	TRUE	TRUE	FALSE	FALSE	TRUE
CHMP2B	ENSG00000083937	ENST00000466696	3	87302198	87303063	+	TRUE	FALSE	TRUE	FALSE	TRUE	FALSE
CLU	ENSG00000120885	ENST00000522413	8	27463898	27472209	−	FALSE	TRUE	TRUE	FALSE	FALSE	TRUE
CNTF	ENSG00000242689	ENST00000361987	11	58390145	58393198	+	TRUE	FALSE	TRUE	FALSE	TRUE	FALSE
CREBBP	ENSG00000005339	ENST00000574740	16	3786508	3794958	−	FALSE	TRUE	TRUE	FALSE	FALSE	TRUE
CST3	ENSG00000101439	ENST00000398409	20	23614293	23619110	−	FALSE	TRUE	TRUE	FALSE	TRUE	FALSE
CTSD	ENSG00000117984	ENST00000438213	11	1775253	1782770	−	FALSE	TRUE	TRUE	FALSE	FALSE	TRUE
DPP6	ENSG00000130226	ENST00000377770	7	153749764	154685161	+	FALSE	TRUE	TRUE	FALSE	TRUE	FALSE
ERBB4	ENSG00000178568	ENST00000484594	2	212426486	213403306	−	TRUE	FALSE	TRUE	FALSE	TRUE	FALSE
FOS	ENSG00000170345	ENST00000556324	14	75745530	75746234	+	FALSE	TRUE	TRUE	FALSE	FALSE	TRUE
GDI1	ENSG00000203879	ENST00000465640	X	153670112	153671075	+	FALSE	TRUE	TRUE	FALSE	FALSE	TRUE
GFAP	ENSG00000131095	ENST00000253408	17	42982993	42992920	−	FALSE	TRUE	TRUE	FALSE	FALSE	TRUE
GLE1	ENSG00000119392	ENST00000309971	9	131266978	131304567	+	FALSE	TRUE	TRUE	FALSE	TRUE	FALSE
GSR	ENSG00000104687	ENST00000221130	8	30535582	30585443	−	FALSE	TRUE	TRUE	FALSE	FALSE	TRUE
GSTP1	ENSG00000084207	ENST00000489040	11	67351604	67352535	+	FALSE	TRUE	TRUE	FALSE	FALSE	TRUE
GSX2	ENSG00000180613	ENST00000326902	4	54966197	54968672	+	FALSE	TRUE	TRUE	FALSE	FALSE	TRUE
HSF1	ENSG00000185122	ENST00000529630	8	145532954	145533780	+	FALSE	TRUE	TRUE	FALSE	FALSE	TRUE
INA	ENSG00000148798	ENST00000369849	10	105036919	105050108	+	FALSE	TRUE	TRUE	FALSE	FALSE	TRUE
JAK3	ENSG00000105639	ENST00000526008	19	17949078	17958841	−	FALSE	TRUE	TRUE	FALSE	FALSE	TRUE
JUND	ENSG00000130522	ENST00000600972	19	18390828	18391739	−	FALSE	TRUE	TRUE	FALSE	FALSE	TRUE
KIF3C	ENSG00000084731	ENST00000455394	2	26149470	26205366	−	FALSE	TRUE	TRUE	FALSE	FALSE	TRUE
LAT	ENSG00000213658	ENST00000566415	16	29000897	29001776	+	FALSE	TRUE	TRUE	FALSE	FALSE	TRUE
LDLR	ENSG00000130164	ENST00000560467	19	11215982	11224300	+	FALSE	TRUE	TRUE	FALSE	FALSE	TRUE
PARK7	ENSG00000116288	ENST00000465354	1	8021807	8031581	+	TRUE	FALSE	TRUE	FALSE	FALSE	FALSE
PLA2G4A	ENSG00000116711	ENST00000466600	1	186823417	186908362	+	FALSE	TRUE	TRUE	FALSE	FALSE	TRUE
PPARGC1A	ENSG00000109819	ENST00000264867	4	23793643	23891700	−	FALSE	TRUE	TRUE	FALSE	FALSE	TRUE
PRPH	ENSG00000135406	ENST00000551194	12	49687034	49687780	+	TRUE	FALSE	TRUE	FALSE	TRUE	FALSE
RXRA	ENSG00000186350	ENST00000484822	9	137208943	137298240	+	FALSE	TRUE	TRUE	FALSE	FALSE	TRUE
SELPLG	ENSG00000110876	ENST00000228463	12	109016604	109025854	−	FALSE	TRUE	TRUE	FALSE	FALSE	TRUE
SHC1	ENSG00000160691	ENST00000448116	1	154934773	154943223	−	FALSE	TRUE	TRUE	FALSE	FALSE	TRUE
SLC1A2	ENSG00000110436	ENST00000531628	11	35287147	35323075	−	FALSE	TRUE	TRUE	FALSE	TRUE	FALSE
SNAI1	ENSG00000124216	ENST00000244050	20	48599535	48605423	+	FALSE	TRUE	TRUE	FALSE	FALSE	TRUE
SOD2	ENSG00000112096	ENST00000541573	6	160103513	160113110	−	FALSE	TRUE	TRUE	FALSE	TRUE	FALSE
TIAM1	ENSG00000156299	ENST00000455508	21	32638611	32716594	−	FALSE	TRUE	TRUE	FALSE	FALSE	TRUE
TLE3	ENSG00000140332	ENST00000557815	15	70341315	70351129	−	FALSE	TRUE	TRUE	FALSE	FALSE	TRUE
TMSB4X	ENSG00000205542	ENST00000451311	X	12993226	12995346	+	FALSE	TRUE	TRUE	FALSE	FALSE	TRUE
TNF	ENSG00000232810	ENST00000449264	6	31543344	31546113	+	FALSE	TRUE	TRUE	FALSE	FALSE	TRUE
TP53	ENSG00000141510	ENST00000574684	17	7577571	7578437	−	FALSE	TRUE	TRUE	FALSE	FALSE	TRUE
TRPM7	ENSG00000092439	ENST00000558444	15	50867155	50874661	−	TRUE	FALSE	TRUE	FALSE	TRUE	FALSE
VIM	ENSG00000026025	ENST00000544301	10	17270257	17279584	+	FALSE	TRUE	TRUE	FALSE	FALSE	TRUE
WNT7A	ENSG00000154764	ENST00000285018	3	13857754	13921618	−	FALSE	TRUE	TRUE	FALSE	FALSE	TRUE
XIAP	ENSG00000101966	ENST00000496602	X	123046609	123047465	+	FALSE	TRUE	TRUE	FALSE	FALSE	TRUE

**Table 3:** Top 20 most enriched Revigo-grouped biological processes (ranked by enrichment fold) for each set of predicted ALS-linked genes. Enrichment was calculated using Pantherdb. All terms are significant at *p*-value < 0.05 level following the Bonferroni correction.

Rank	DisGeNet	ALSoD	Manual	ClinVar	Union
1	Smooth muscle contraction	Peripheral nervous system development	Erythrocyte differentiation	Cardiac muscle tissue development	Peripheral nervous system development
2	Response to xenobiotic stimulus	Response to xenobiotic stimulus	Translational termination	Nuclear migration	Protein localization to Golgi apparatus
3	Response to antibiotic	Phosphatidylcholine metabolic process	Phospholipid catabolic process	Nucleus localization	Response to antibiotic
4	Tissue remodelling	Inactivation of MAPK activity	Regulation of transcription from RNA polymerase II promoter in response to stress	Heart contraction	Regulation of neuron death
5	Sprouting angiogenesis	Ammonium transport	Response to heat	Cellular component assembly involved in morphogenesis	Mitochondrial fusion
6	Peripheral nervous system development	Protein localization to Golgi apparatus	Endosome transport via multivesicular body sorting pathway (GO:0032509)	Myofibril assembly	Response to mechanical stimulus
7	Response to hypoxia	Response to antibiotic	Multivesicular body sorting pathway	Response to mechanical stimulus	Regulation of phosphatidylinositol 3-kinase signalling
8	Regulation of blood pressure	Triglyceride homeostasis	Glutathione metabolic process	Skeletal muscle contraction	Ammonium transport
9	Ammonium transport	Behaviour	Regulation of cysteine-type endopeptidase activity	Protein homooligomerization	Regulation of phospholipase activity
10	Regulation of phospholipase activity	Ammonium ion metabolic process	Cellular modified amino acid metabolic process	Muscle structure development	Regulation of lipase activity
11	Phospholipase C-activating G protein-coupled receptor signalling pathway	Positive regulation of lipase activity	Wnt signalling pathway	Cellular lipid catabolic process	Protein targeting to the vacuole
12	Regulation of mitochondrion organization	Organophosphate ester transport	Apoptotic process	Synapse organization	Phospholipase C-activating G protein-coupled receptor signalling pathway
13	Positive regulation of protein kinase B signalling	Phospholipid transport	Cell death	Lipid catabolic process	Positive regulation of blood pressure
14	Response to oxidative stress	Phospholipase C-activating G protein-coupled receptor signalling pathway	Sulfur compound metabolic process	Circulatory system process	Action potential
15	Regulation of lipase activity	Positive regulation of the cellular catabolic process	Response to abiotic stimulus	Actomyosin structure organization	Membrane protein proteolysis
16	Superoxide metabolic process	Regulation of neurotransmitter levels	Positive regulation of DNA-binding transcription factor activity	Cell-cell adhesion via plasma-membrane adhesion molecules	Behaviour
17	Membrane protein proteolysis	Regulation of trans-synaptic signalling	Response to cytokine	Regulation of canonical Wnt signalling pathway	Protein localization to the vacuole
18	Behaviour	Regulation of lipase activity	Positive regulation of molecular function	Negative regulation of the apoptotic process	Vacuolar transport
19	Glycolytic process	Glutamate receptor signalling pathway	Cell surface receptor signalling pathway	Circulatory system development	Ammonium ion metabolic process
20	Cyclic nucleotide metabolic process	Response to drug	Regulation of molecular function	Maintenance of location	Regulation of trans-synaptic signalling

**Table 4:** Top 5 most enriched human diseases in Enrichr analysis based on the OMIM disease database for each set of predicted ALS-linked genes. Overlapping genes are between brackets. Only diseases significant at adjusted *p*-value < 0.05 are reported.

Rank	DisGeNet	ALSoD	Manual	ClinVar	Union
1	Parkinson disease (PRKN;NR4A2;PINK1;UCHL1;TBP;HTRA2;MAPT;SNCAIP;FBXO7;SNCA)	Parkinson disease (PRKN;NR4A2;PINK1;UCHL1;TBP;HTRA2;DBH;SNCAIP;FBXO7;SNCA)	Amyotrophic lateral sclerosis (ALS2;CHMP2B;TRPM7;PRPH)	Cardiomyopathy(DSP;MYBPC3;CAV3;ACTN2;TPM1;LDB3;ABCC9;PSEN1;TAZ;TTN;PLN;SGCD;DES;ACTC1;MYL2;LMNA;MYL3;TCAP;TNNI3;DMD;SCN5A;MYH6;VCL;MYH7)	Cardiomyopathy (DSP;MYBPC3;CAV3;ACTN2;TPM1;PSEN2;LDB3;ABCC9;TAZ;TTN;PLN;SGCD;DES;ACTC1;MYL2;LMNA;MYL3;TCAP;TNNI3;DMD;SCN5A;MYH6;VCL;MYH7)
2	Dystonia (SGCE;GCH1;PRKRA;ATP1A3;DRD2;THAP1;TAF1)	Alzheimer′s disease (APP;NOS3;PSEN2;APBB2;BLMH;A2M;MPO;SORL1)	Frontotemporal dementia (CHMP2B;TRPM7;TNF)	Cardiomyopathy, dilated (DSP;MYBPC3;ACTN2;TPM1;LDB3;ABCC9;PSEN1;TAZ;TTN;PLN;SGCD;DES;ACTC1;LMNA;TCAP;TNNI3;DMD;SCN5A;VCL;MYH7)	Cardiomyopathy, dilated (DSP;MYBPC3;ACTN2;TPM1;PSEN2;LDB3;ABCC9;TAZ;TTN;PLN;SGCD;DES;ACTC1;LMNA;TCAP;TNNI3;DMD;SCN5A;VCL;MYH7)
3	Diabetes (IL6;EPO;IRS1;HFE;INSR;IRS2;PPARG;SLC2A4;GCK)	Dystonia (SGCE;GCH1;PRKRA;ATP1A3;DRD2;THAP1;TAF1)		Charcot-Marie-Tooth disease (PRPS1;MTMR2;EGR2;HSPB8;LITAF;NDRG1;DNM2;MPZ;LMNA;MFN2;NEFL;KIF1B;GARS;SBF2)	Ataxia (PRKCG;TBP;ABCB7;FMR1;KCNA1;ITPR1;SLC1A3;CP;APTX;SYNE1;TTBK2;ATCAY;CACNB4;ATXN1;ATXN7;PPP2R2B;TDP1;SACS;ATXN10;FXN;POLG;SPTBN2)
4	Diabetes mellitus, type 2 (IRS1;INSR;IRS2;PPARG;SLC2A4;GCK)	Schizophrenia (CHRNA7;DTNBP1;AKT1;MTHFR;NRG1;HTR2A;COMT)		Neuropathy (EGR2;CTDP1;HSPB8;BSCL2;SPTLC1;GAN;WNK1;MPZ;TDP1;PRX;MFN2;GARS;CCT5;POLG)	Charcot-Marie-Tooth disease (PRPS1;MTMR2;EGR2;HSPB8;LITAF;NDRG1;DNM2;MPZ;LMNA;MFN2;NEFL;KIF1B;SBF2)
5	Alzheimer disease (NOS3;HFE;PSEN1;MPO)	Colorectal cancer (PLA2G2A;AKT1;BAX;CTNNB1;TLR4;TP53)		Cardiomyopathy, hypertrophic (MYBPC3;ACTC1;CAV3;MYL2;TPM1;MYL3;TNNI3;MYH6;TTN;MYH7)	Cardiomyopathy, hypertrophic (MYBPC3;ACTC1;CAV3;MYL2;TPM1;MYL3;TNNI3;MYH6;TTN;MYH7)

**Table 5:** Validation of predicted ALS-linked genes. *ZFP91-CNTF is a read-through transcript of both *ZPF91* and *CNTF*. *CNTF* was predicted to be ALS-linked by the model. The *p*-value and number of validated genes shown in brackets indicate results if the *ZFP91-CNTF* transcript is not considered.

	*p*-Value	Predicted (n)	Mapped (n)	Validated (n)	Significant genes (*p*-Value)	Magma Gene set *p*-Value
DisGeNet	0.49	176	166	1	*ATXN3* (6.8 × 10^−7^)	0.44
ALSoD	0.33	327	305	2	*SCFD1* (5.4 × 10^−7^) *ATXN3* (6.8 × 10^−7^)	0.24
Manual	0.060 (0.33)	45	41	2 (1)	*WNT7A* (0.00019) ZFP91-CNTF* (0.00091)	0.057
ClinVar	0.038	192	170	3	*WNT7A* (0.00019) *SCFD1*(5.4 × 10^−7^) *UNC13A* (2.9 × 10^−6^)	0.065
Union	0.67	575	534	1	*ATXN3* (6.8 × 10^−7^)	0.72

## Supplementary Materials

The following are available online at https://www.mdpi.com/2073-4425/11/6/668/s1, Supplementary Figure S1: GWAS association signal of the *ZFP91-CNTF* locus; Supplementary File 1: All sets of known ALS-linked genes; Supplementary File 2: All sets of predicted ALS-linked genes; Supplementary File 3: functional enrichment analysis results.

## References

[1] Brown R.H., Al-Chalabi A. (2017). Amyotrophic lateral sclerosis. N. Engl. J. Med..

[2] Abel O., Powell J.F., Andersen P.M., Al-Chalabi A. (2012). ALSoD: A user-friendly online bioinformatics tool for amyotrophic lateral sclerosis genetics. Hum. Mutat..

[3] Iacoangeli A., Al Khleifat A., Sproviero W., Shatunov A., Jones A.R., Opie-Martin S., Naselli E., Topp S.D., Fogh I., Hodges A. (2019). ALSgeneScanner: A pipeline for the analysis and interpretation of DNA sequencing data of ALS patients. Amyotroph. Lateral Scler. Front. Degener..

[4] Renton A.E., Chiò A., Traynor B.J. (2014). State of play in amyotrophic lateral sclerosis genetics. Nat. Neurosci..

[5] Chia R., Chiò A., Traynor B.J. (2018). Novel genes associated with amyotrophic lateral sclerosis: Diagnostic and clinical implications. Lancet Neurol..

[6] Al-Chalabi A. (2017). Perspective: Don’t keep it in the family. Nature.

[7] Stelzl U., Wanker E.E. (2006). The value of high quality protein–protein interaction networks for systems biology. Curr. Opin. Chem. Biol..

[8] Piñero J., Bravo À., Queralt-Rosinach N., Gutiérrez-Sacristán A., Deu-Pons J., Centeno E., García-García J., Sanz F., Furlong L.I. (2016). DisGeNET: A comprehensive platform integrating information on human disease-associated genes and variants. Nucleic Acids Res..

[9] Iacoangeli A., Al Khleifat A., Sproviero W., Shatunov A., Jones A., Morgan S., Pittman A., Dobson R., Newhouse S., Al-Chalabi A. (2019). DNAscan: Personal computer compatible NGS analysis, annotation and visualisation. BMC Bioinform..

[10] Gandhi T., Zhong J., Mathivanan S., Karthick L., Chandrika K., Mohan S.S., Sharma S., Pinkert S., Nagaraju S., Periaswamy B. (2006). Analysis of the human protein interactome and comparison with yeast, worm and fly interaction datasets. Nat. Genet..

[11] Oti M., Brunner H.G. (2007). The modular nature of genetic diseases. Clin. Genet..

[12] Lin C.-H., Konecki D.M., Liu M., Wilson S.J., Nassar H., Wilkins A.D., Gleich D.F., Lichtarge O. (2019). Multimodal network diffusion predicts future disease–gene–chemical associations. Bioinformatics.

[13] Köhler S., Bauer S., Horn D., Robinson P.N. (2008). Walking the interactome for prioritization of candidate disease genes. Am. J. Hum. Genet..

[14] Peng J., Bai K., Shang X., Wang G., Xue H., Jin S., Cheng L., Wang Y., Chen J. (2017). Predicting disease-related genes using integrated biomedical networks. BMC Genom..

[15] Vanunu O., Magger O., Ruppin E., Shlomi T., Sharan R. (2010). Associating genes and protein complexes with disease via network propagation. PLoS Comput. Biol..

[16] Zhou H., Skolnick J. (2016). A knowledge-based approach for predicting gene–disease associations. Bioinformatics.

[17] Zeng X., Liao Y., Liu Y., Zou Q. (2016). Prediction and validation of disease genes using HeteSim Scores. IEEE/ACM Trans. Comput. Biol. Bioinform..

[18] Ata S.K., Ou-Yang L., Fang Y., Kwoh C.-K., Wu M., Li X.-L. (2018). Integrating node embeddings and biological annotations for genes to predict disease-gene associations. BMC Syst. Biol..

[19] Grover A., Leskovec J. (2016). node2vec: Scalable feature learning for networks. Proceedings of the 22nd ACM SIGKDD International Conference on Knowledge Discovery and Data Mining.

[20] Wu X., Jiang R., Zhang M.Q., Li S. (2008). Network-based global inference of human disease genes. Mol. Syst. Biol..

[21] Lin L., Yang T., Fang L., Yang J., Yang F., Zhao J. (2017). Gene gravity-like algorithm for disease gene prediction based on phenotype-specific network. BMC Syst. Biol..

[22] Luo P., Li Y., Tian L.-P., Wu F.-X. (2019). Enhancing the prediction of disease–gene associations with multimodal deep learning. Bioinformatics.

[23] Asif M., Martiniano H.F., Vicente A.M., Couto F.M. (2018). Identifying disease genes using machine learning and gene functional similarities, assessed through Gene Ontology. PLoS ONE.

[24] Bean D.M., Wu H., Iqbal E., Dzahini O., Ibrahim Z.M., Broadbent M., Stewart R., Dobson R.J. (2017). Knowledge graph prediction of unknown adverse drug reactions and validation in electronic health records. Sci. Rep..

[25] Orchard S., Ammari M., Aranda B., Breuza L., Briganti L., Broackes-Carter F., Campbell N.H., Chavali G., Chen C., Del-Toro N. (2014). The MIntAct project—IntAct as a common curation platform for 11 molecular interaction databases. Nucleic Acids Res..

[26] Dolinski K., Dwight S.S., Eppig J.T., Harris M.A., Hill D.P., Issel-Tarver L., Kasarskis A., Lewis S., Matese J., The Gene Ontology Consortium (2000). Gene ontology: Tool for the unification of biology. Nat. Genet..

[27] Acencio M.L., Lægreid A., Kuiper M. (2019). The Gene Ontology Resource: 20 Years and Still Going Strong. Nucleic Acids Res..

[28] Piñero J., Ramírez-Anguita J.M., Saüch-Pitarch J., Ronzano F., Centeno E., Sanz F., Furlong L.I. (2020). The DisGeNET knowledge platform for disease genomics: 2019 Update. Nucleic Acids Res..

[29] Wroe R., Wai-Ling Butler A., Andersen P.M., Powell J.F., Al-Chalabi A. (2008). ALSOD: The Amyotrophic Lateral Sclerosis Online Database. Amyotroph. Lateral Scler..

[30] Landrum M.J., Lee J.M., Riley G.R., Jang W., Rubinstein W.S., Church D.M., Maglott D.R. (2014). ClinVar: Public archive of relationships among sequence variation and human phenotype. Nucleic Acids Res..

[31] Mi H., Muruganujan A., Ebert D., Huang X., Thomas P.D. (2019). PANTHER version 14: More genomes, a new PANTHER GO-slim and improvements in enrichment analysis tools. Nucleic Acids Res..

[32] Supek F., Bošnjak M., Škunca N., Šmuc T. (2011). REVIGO summarizes and visualizes long lists of gene ontology terms. PLoS ONE.

[33] Kuleshov M.V., Jones M.R., Rouillard A.D., Fernandez N.F., Duan Q., Wang Z., Koplev S., Jenkins S.L., Jagodnik K.M., Lachmann A. (2016). Enrichr: A comprehensive gene set enrichment analysis web server 2016 update. Nucleic Acids Res..

[34] Amberger J.S., Bocchini C.A., Scott A.F., Hamosh A. (2019). Omim. org: Leveraging knowledge across phenotype–gene relationships. Nucleic Acids Res..

[35] Hamosh A., Scott A.F., Amberger J.S., Bocchini C.A., McKusick V.A. (2005). Online Mendelian Inheritance in Man (OMIM), a knowledgebase of human genes and genetic disorders. Nucleic Acids Res..

[36] Nicolas A., Kenna K.P., Renton A.E., Ticozzi N., Faghri F., Chia R., Dominov J.A., Kenna B.J., Nalls M.A., Keagle P. (2018). Genome-wide analyses identify KIF5A as a novel ALS gene. Neuron.

[37] Pe’er I., Yelensky R., Altshuler D., Daly M.J. (2008). Estimation of the multiple testing burden for genomewide association studies of nearly all common variants. Genet. Epidemiol. Off. Publ. Int. Genet. Epidemiol. Soc..

[38] The 1000 Genomes Project Consortium (2015). A global reference for human genetic variation. Nature.

[39] de Leeuw C.A., Mooij J.M., Heskes T., Posthuma D. (2015). MAGMA: Generalized gene-set analysis of GWAS data. PLoS Comput. Biol..

[40] Oosthuyse B., Moons L., Storkebaum E., Beck H., Nuyens D., Brusselmans K., Van Dorpe J., Hellings P., Gorselink M., Heymans S. (2001). Deletion of the hypoxia-response element in the vascular endothelial growth factor promoter causes motor neuron degeneration. Nat. Genet..

[41] Adibhatla R.M., Hatcher J.F. (2007). Role of lipids in brain injury and diseases. Future Lipidol..

[42] Smith E.F., Shaw P.J., De Vos K.J. (2019). The role of mitochondria in amyotrophic lateral sclerosis. Neurosci. Lett..

[43] Guo W., Vandoorne T., Steyaert J., Staats K.A., Van Den Bosch L. (2020). The multifaceted role of kinases in amyotrophic lateral sclerosis: Genetic, pathological and therapeutic implications. Brain.

[44] Barber S.C., Shaw P.J. (2010). Oxidative stress in ALS: Key role in motor neuron injury and therapeutic target. Free Radic. Biol. Med..

[45] Bowling A.C., Schulz J.B., Brown R.H., Beal M.F. (1993). Superoxide dismutase activity, oxidative damage, and mitochondrial energy metabolism in familial and sporadic amyotrophic lateral sclerosis. J. Neurochem..

[46] Nishimura A.L., Mitne-Neto M., Silva H.C., Richieri-Costa A., Middleton S., Cascio D., Kok F., Oliveira J.R., Gillingwater T., Webb J. (2004). A mutation in the vesicle-trafficking protein VAPB causes late-onset spinal muscular atrophy and amyotrophic lateral sclerosis. Am. J. Hum. Genet..

[47] Foerster B.R., Pomper M.G., Callaghan B.C., Petrou M., Edden R.A., Mohamed M.A., Welsh R.C., Carlos R.C., Barker P.B., Feldman E.L. (2013). An imbalance between excitatory and inhibitory neurotransmitters in amyotrophic lateral sclerosis revealed by use of 3-T proton magnetic resonance spectroscopy. JAMA Neurol..

[48] Lillo P., Mioshi E., Zoing M.C., Kiernan M.C., Hodges J.R. (2011). How common are behavioural changes in amyotrophic lateral sclerosis?. Amyotroph. Lateral Scler..

[49] DeJesus-Hernandez M., Mackenzie I.R., Boeve B.F., Boxer A.L., Baker M., Rutherford N.J., Nicholson A.M., Finch N.A., Flynn H., Adamson J. (2011). Expanded GGGGCC hexanucleotide repeat in noncoding region of C9ORF72 causes chromosome 9p-linked FTD and ALS. Neuron.

[50] Renton A.E., Majounie E., Waite A., Simón-Sánchez J., Rollinson S., Gibbs J.R., Schymick J.C., Laaksovirta H., Van Swieten J.C., Myllykangas L. (2011). A hexanucleotide repeat expansion in C9ORF72 is the cause of chromosome 9p21-linked ALS-FTD. Neuron.

[51] McLaughlin R.L., Schijven D., Van Rheenen W., Van Eijk K.R., O’Brien M., Kahn R.S., Ophoff R.A., Goris A., Bradley D.G., Al-Chalabi A. (2017). Genetic correlation between amyotrophic lateral sclerosis and schizophrenia. Nat. Commun..

[52] Trist B.G., Davies K.M., Cottam V., Genoud S., Ortega R., Roudeau S., Carmona A., De Silva K., Wasinger V., Lewis S.J. (2017). Amyotrophic lateral sclerosis-like superoxide dismutase 1 proteinopathy is associated with neuronal loss in Parkinson’s disease brain. Acta Neuropathol..

[53] Muraoka Y., Nakamura A., Tanaka R., Suda K., Azuma Y., Kushimura Y., Piccolo L.L., Yoshida H., Mizuta I., Tokuda T. (2018). Genetic screening of the genes interacting with Drosophila FIG4 identified a novel link between CMT-causing gene and long noncoding RNAs. Exp. Neurol..

[54] Montecchiani C., Pedace L., Lo Giudice T., Casella A., Mearini M., Gaudiello F., Pedroso J.L., Terracciano C., Caltagirone C., Massa R. (2016). ALS5/SPG11/KIAA1840 mutations cause autosomal recessive axonal Charcot–Marie–Tooth disease. Brain.

[55] Orlacchio A., Babalini C., Borreca A., Patrono C., Massa R., Basaran S., Munhoz R.P., Rogaeva E.A., St George-Hyslop P.H., Bernardi G. (2010). SPATACSIN mutations cause autosomal recessive juvenile amyotrophic lateral sclerosis. Brain.

[56] Zeng P., Wang T., Zheng J., Zhou X. (2019). Causal association of type 2 diabetes with amyotrophic lateral sclerosis: New evidence from Mendelian randomization using GWAS summary statistics. BMC Med..

[57] Kioumourtzoglou M.-A., Rotem R.S., Seals R.M., Gredal O., Hansen J., Weisskopf M.G. (2015). Diabetes mellitus, obesity, and diagnosis of amyotrophic lateral sclerosis: A population-based study. JAMA Neurol..

[58] Miller R.G., Mitchell J.D., Moore D.H. (2012). Riluzole for amyotrophic lateral sclerosis (ALS)/motor neuron disease (MND). Cochrane Database Syst. Rev..

[59] Crockford C., Newton J., Lonergan K., Chiwera T., Booth T., Chandran S., Colville S., Heverin M., Mays I., Pal S. (2018). ALS-specific cognitive and behavior changes associated with advancing disease stage in ALS. Neurology.

[60] Phukan J., Pender N.P., Hardiman O. (2007). Cognitive impairment in amyotrophic lateral sclerosis. Lancet Neurol..

[61] van Es M.A., Hardiman O., Chio A., Al-Chalabi A., Pasterkamp R.J., Veldink J.H., van den Berg L.H. (2017). Amyotrophic lateral sclerosis. Lancet.

[62] Van Rheenen W., Shatunov A., Dekker A.M., McLaughlin R.L., Diekstra F.P., Pulit S.L., Van Der Spek R.A., Võsa U., De Jong S., Robinson M.R. (2016). Genome-wide association analyses identify new risk variants and the genetic architecture of amyotrophic lateral sclerosis. Nat. Genet..

[63] Fogh I., Ratti A., Gellera C., Lin K., Tiloca C., Moskvina V., Corrado L., Sorarù G., Cereda C., Corti S. (2013). A genome-wide association meta-analysis identifies a novel locus at 17q11. 2 associated with sporadic amyotrophic lateral sclerosis. Hum. Mol. Genet..

[64] Fogh I., Lin K., Tiloca C., Rooney J., Gellera C., Diekstra F.P., Ratti A., Shatunov A., Van Es M.A., Proitsi P. (2016). Association of a locus in the CAMTA1 gene with survival in patients with sporadic amyotrophic lateral sclerosis. JAMA Neurol..

[65] Iacoangeli A., Al Khleifat A., Jones A.R., Sproviero W., Shatunov A., Opie-Martin S., Morrison K.E., Shaw P.J., Shaw C.E., Fogh I. (2019). C9orf72 intermediate expansions of 24–30 repeats are associated with ALS. Acta Neuropathol. Commun..

[66] Project MinE ALS Sequencing Consortium (2018). Project MinE: Study design and pilot analyses of a large-scale whole-genome sequencing study in amyotrophic lateral sclerosis. Eur. J. Hum. Genet..

[67] Kenna K.P., Van Doormaal P.T., Dekker A.M., Ticozzi N., Kenna B.J., Diekstra F.P., Van Rheenen W., Van Eijk K.R., Jones A.R., Keagle P. (2016). NEK1 variants confer susceptibility to amyotrophic lateral sclerosis. Nat. Genet..

[68] Van Rheenen W., Diekstra F.P., Harschnitz O., Westeneng H.-J., van Eijk K.R., Saris C.G., Groen E.J., Van Es M.A., Blauw H.M., Van Vught P.W. (2018). Whole blood transcriptome analysis in amyotrophic lateral sclerosis: A biomarker study. PLoS ONE.

[69] van der Spek R.A., Van Rheenen W., Pulit S.L., Kenna K.P., van den Berg L.H., Veldink J.H., On behalf of the Project MinE ALS Sequencing Consortium (2019). The Project MinE databrowser: Bringing large-scale whole-genome sequencing in ALS to researchers and the public. Amyotroph. Lateral Scler. Front. Degener..

[70] Farhan S.M., Howrigan D.P., Abbott L.E., Klim J.R., Topp S.D., Byrnes A.E., Churchhouse C., Phatnani H., Smith B.N., Rampersaud E. (2019). Exome sequencing in amyotrophic lateral sclerosis implicates a novel gene, DNAJC7, encoding a heat-shock protein. Nat. Neurosci..

[71] Al Khleifat A., Iacoangeli A., Shatunov A., Fang T., Sproviero W., Jones A.R., Opie-Martin S., Morrison K.E., Shaw P.J., Shaw C.E. (2019). Telomere length is greater in ALS than in controls: A whole genome sequencing study. Amyotroph. Lateral Scler. Front. Degener..

