# Supplementary material for: A Knowledge-Based Machine Learning Approach to Gene Prioritisation in Amyotrophic Lateral Sclerosis
Source: Genes (Basel). 2020 Jun 19;11(6):668. doi: 10.3390/genes11060668 (PMC7349022; doi:10.3390/genes11060668)
Supplement: Supplementary file 1 [file genes-11-00668-s001.zip › supplementary_materials-4/Supplementary Figure S1.docx]

Figure S1. GWAS association signal of the *ZFP91-CNTF* locus.
